# Supplementary material for: The stability of subducted glaucophane with the Earth’s secular cooling
Source: Nat Commun. 2021 Mar 5;12:1496. doi: 10.1038/s41467-021-21746-8 (PMC7935898; doi:10.1038/s41467-021-21746-8)
Supplement: Supplementary file 1 — Supplementary Information [file 41467_2021_21746_MOESM1_ESM.pdf]

Supplementary Information

The stability of subducted glaucophane with the Earth's secular cooling

Yoonah Bang[1], Huijeong Hwang[1], Taehyun Kim[1], Hyunchae Cynn[2], Yong Park[3], Haemyeong Jung[3], Changyong Park[4], Dmitry Popov[4], Vitali B. Prakapenka[5], Lin Wang[6], Hanns-Peter Liermann[7], Tetsuo Irifune[8], Ho-Kwang Mao[6] and Yongjae Lee[1, *]

[1] *Department of Earth System Sciences, Yonsei University, Seoul 03722, South Korea*
[2] *Physics Division, Physical and Life Sciences Directorate, Lawrence Livermore National Laboratory, Livermore, CA 94550, USA*
[3] *School of Earth and Environmental Sciences, Seoul National University, Seoul 08826, South Korea*
[4] *High Pressure Collaborative Access Team, X-ray Science Division, Argonne National Laboratory, Argonne, IL 60439, USA*
[5] *Center for Advanced Radiation Sources, University of Chicago, Argonne, IL 60439, USA*
[6] *Center for High Pressure Science & Technology Advanced Research, Shanghai 201203, China*
[7] *Photon Sciences, Deutsches Elektronen-Synchrotron (DESY), Hamburg 22607, Germany,*
[8] *Geodynamics Research Center, Ehime University, Matsuyama, Ehime 790-8577, Japan*

Supplementary Methods

1. Chemical analysis and X-ray diffraction on glaucophane at ambient conditions

The glaucophane sample used in our diamond-anvil cell (DAC) experiments is from the glaucophanite outcrops along Rio Oremo in Piedmonte, Italy. Elemental analysis on the glaucophane sample was carried out using a low-vacuum SEM (JCM-5000 Neoscope^{TM}) and an EDS system (Oxford Instruments) at Yonsei University, Korea (Supplementary Fig. 1). The derived chemical formula of the glaucophane is $Na_{2.09}(Mg_{2.7}Fe_{0.8}Al_{1.6})Si_{7.97}O_{22}(OH)_2$. Synchrotron X-ray powder diffraction measurement on the glaucophane sample was performed at ambient conditions at beamline 3D-XRS at Pohang Light Source-II (PLS-II). The X-ray beam from the bending magnet was tuned to a wavelength of 0.6888(1) Å (18 keV) and beam size of 100 x 100 μm^2. A Mar345 image plate detector was used to collect diffraction data at a distance of 315.7 mm from the sample with a 200 sec exposure time. Two-dimensional intensity data were converted into one-dimensional data using the Fit2D program[1], which were then analyzed using the Rietveld method as implemented in the GSAS suite of programs[2,3]. Glaucophane was indexed to space group C2/m with the refined cell parameters of a=9.5271(3) Å, b=17.7085(6) Å, c=5.2867(2) Å, β=103.683(2)° (Supplementary Fig. 1 and Supplementary Table 1).

2. Synchrotron X-ray powder diffraction experiments using resistive-heated DAC (RH-DAC)

In-situ high-pressure and high-temperature synchrotron X-ray powder diffraction experiments using a RH-DAC were carried out at beamline 16-BM-D of HPCAT at the Advanced Photon Source (APS) at Argonne National Laboratory and the Extreme Conditions Beamline (ECB) P02.2 at PETRA III. At beamline 16-BM-D, the X-ray beam from the bending magnet was tuned to a wavelength of 0.6199(1) Å (20 KeV) or 0.4959(1) Å (25 KeV) and collimated to 5 x 6 μm^2 in size (FWHM) using a KB-mirror. A Mar345 imaging plate detector was used to collect diffraction data at a distance of 398.7 mm or 379.7 mm from the sample with 120 sec exposure time. At beamline P02.2, the X-ray beam from the undulator source was tuned to a wavelength of 0.4828(1) Å (25.68 keV) and collimated to 8 x 2 μm^2 in size (FWHM) using Compound Refractive Lense (CRL) optics. A Perkin Elmer XRD 1621 detector was used to collect diffraction data at a distance of 401 mm from the sample with 10 sec exposure time. A symmetric-type DAC with culet diameter of 500 μm (or 300 μm at P02.2) was used as a high-pressure vessel. A rhenium gasket of 250 μm thickness was indented to 80 μm (or 50 μm) , and a hole of 250 μm (or 150 μm) in diameter was drilled in the center as a sample chamber using an Electric Discharge Machine (EDM)[4]. Powdered sample of glaucophane was compressed into a thin flake of approximately 50-60 μm in thickness, and then loaded into the gasket hole together with pressure marker such as Au or Cu between layers of pre-baked NaCl powder foils to be used as both a pressure-transmitting medium (PTM) in dry condition and thermal insulator for the sample. A few ruby spheres were placed in the gasket hole as additional pressure marker to be used for the ruby fluorescent method[5]. We carried out additional in-situ P-T experiments by adding ca. 4-5 wt.% H_2O in the glaucophane powder sample to simulate wet environment relevant to certain subduction P-T conditions (Fig. 1).

Simultaneous P-T condition was created by using a resistive coil heater surrounding each diamond-anvil. Two types of coil heaters were used with one set made of a bundle of molybdenum wires of 0.23 mm in diameter each with total 3.5 Ω resistance and the other set made of a platinum

wire of 0.28 mm in diameter with 0.8 Ω resistance for the slab Moho and slab surface conditions, respectively. The coils were covered with alumina oxide paste. During the experiment we followed the P-T conditions of the subduction models to cover separately the slab surface and Moho conditions[6] (Supplementary Table 2). The temperature was monitored using a K-type thermocouple to the maximum uncertainties of ±3 ºC, and the pressure was calculated using the equation of state of the Au or Cu pressure marker[7]. At beamline P02.2, graphite foil heater was used with 0.7 Ω resistance[8]. The detailed description of this type of RH-DAC can be found in the literature[8,9]. Initially, we compressed the sample to 0.5 GPa and then increased the temperature and pressure in increments of 50 ºC and 0.5 GPa up to 450±30 ºC and 5.6(3) GPa for slab Moho and 760±45 ºC and 7.8(3) GPa for slab surface of the cold subduction model.

3. Synchrotron X-ray powder diffraction experiments using laser-heated DAC (LH-DAC)

In-situ high-pressure and high-temperature synchrotron X-ray powder diffraction experiments using a double-sided LH-DAC were conducted at beamline 13-ID-D of GSECARS at the APS. The X-ray beam from the undulator source was tuned to a wavelength of 0.3344(1) Å (37 keV) and collimated to 3 x 4 μm^2 in size (FWHM) using a KB-mirror. Two near-infrared fiber laser beams focused to a flat top of ~10 μm in diameter on sample and up to 100 W power were precisely aligned with the X-ray beam on both sides of the sample[10]. A PILATUS 3X CdTe 1M pixel array detector was used to collect diffraction data. XRD images were integrated and analyzed using the DIOPTAS software[11]. A rigid foil of glaucophane sample was made by compressing the powder to a thickness of 25 μm. The disk was then loaded into a 250 μm diameter hole of a Re gasket pre-indented to 60 μm thickness. The glaucophane foil was propped by 3 or 4 spacer foils of the same material before filling Ne gas as PTM, which is necessary to reduce thermal gradients during laser heating. A few ruby chips were also loaded in the gasket hole as pressure marker[5].

The sample was initially pressurized to 2.9(1) GPa for slab Moho and 5.5(1) GPa for slab surface conditions. At each pressure, temperature was increased by tuning the laser power. The sample temperature was estimated by measuring thermal emission spectra from both sides of the sample and by fitting the spectra to the Planck radiation function[12], which led to the maximum uncertainties of ±100 ºC.

Additional laser-heating experiment was performed ex-situ using the laser heating system at the Center for High Pressure Science and Technology Advanced Research (HPSTAR), China, which consists of two ytterbium fiber lasers and spectrometers to provide double-sided heating and temperature measurement. Sample was prepared in the same way as the LH-DAC experiment at the APS (13-ID-D). For the Proterozoic tectonic setting with the high thermal gradients conditions, the sample was compressed to 1.4(1) GPa and heated up to 1390±30 ºC for 3 minutes using laser power of 1/8 E_0 and beam size of 30 x 30 μm^2. The laser-heated spots were measured by X-ray diffraction at the 16-BM-D beamline at APS.

4. Synchrotron X-ray powder diffraction experiments using external heating of DAC in furnace

Ex-situ high-pressure and high-temperature X-ray powder diffraction experiments on glaucophane were carried out at the Extreme Conditions Beamline (ECB) P02.2 at PETRA-III, beamline 5A (MS-XRS) at PLS-II, and the Institute of High-Pressure Mineral Physics and Chemistry at Yonsei University equipped with a custom-built micro-X-ray diffractometer. At beamline P02.2,

the X-ray beam from the undulator source was tuned to a wavelength of 0.4838(1) Å (25.6 keV) and collimated to 8 x 2 μm^2 in size (FWHM) using CRL optics. A Perkin Elmer XRD 1621 detector was used to collect diffraction data at a distance of 392 mm from the sample with 10 sec exposure time. At beamline 5A (MS-XRS), the X-ray beam from the undulator was tuned to a wavelength of 0.6924(1) Å (17.9 keV) and collimated to 100 x 100 μm^2 in size. A Mar345 image plate detector was used to collect diffraction data at a distance of 285 mm from the sample with 300 sec exposure time. At Yonsei University, monochromatic X-ray (Mo-Kα of 0.7107 Å, Rigaku MicroMax-007HF) with beam size of 200 x 200 μm^2 was used to collect diffraction data using an R-axis IV^{++} imaging plate detector at a distance of 150 mm from the sample with 600 sec exposure time.

The sample was compressed up to 3.3(1) GPa while the temperature was applied ex-situ by placing the DAC in a furnace for ca. 1-2 hours up to 500 ºC and 560 ºC for the cold and warm slab surface conditions, respectively.

5. Multi-anvil press experiment on glaucophane and FT-IR measurement

High-pressure and high-temperature synthesis experiment on glaucophane was performed to investigate the breakdown product using a 1000-ton multi-anvil press (Orange-1000) at the Geodynamics Research Center at Ehime University[13]. We employed a 2.5 mm outer diameter gold capsule of 3 mm length and 0.1 mm wall thickness. After loading the glaucophane powder, gold foils were inserted at the top of the sample, which was then flattened to have a uniform thickness from the top to the bottom. Sintered (Mg,Co)O octahedral pressure media with 11 mm edge length and a graphite heater were used in the sample assembly for the P-T experiment. Pressure was applied by 8 tungsten carbide cubic anvils with 11 mm truncated edge lengths. Pressure was increased up to 130 ton, equivalent to 3.0 GPa with the uncertainties of ±0.5 GPa, followed by temperature increase to 950 ºC, which was held for 3 hours while monitoring temperature with a K-type thermocouple to the maximum uncertainties of ±5 ºC (Supplementary Fig. 5). After the experiment, the capsule was retrieved from the sintered octahedron and then cut open to recover the sample. The run product displayed a changed in color, from pale blue to black, similar to the decolorization observed in the LH-DAC experiment.

Fourier-transform infrared spectroscopy (FT-IR) measurement was performed on the original and the recovered sample from the multi-anvil press experiment to investigate the changes in the O-H stretching modes (Supplementary Fig. 6 and Supplementary Table 3). Both samples were mixed separately with KBr powder in a 1:50 weight ratio and ground in an agate mortar. Each mixed sample was baked for 30-60 min at 80 ºC to eliminate absorbed water and then compressed into a pellet before measuring IR spectra. FT-IR spectra were collected using the custom-built IR setup at the Institute of High-Pressure Mineral Physics and Chemistry at Yonsei University, equipped with a Bruker TENSOR II spectrometer, a liquid N_2 cooled MCT detector, and KBr beam splitter for MIR source (400-4000 cm^{-1}). Total 512 scans were accumulated on each sample to have the spectral resolution of ca. 4 cm^{-1}.

6. A modified Griggs apparatus experiment on natural blueschist rock and SEM-EDS measurement

The starting material was a natural epidote blueschist rock collected from Voltri massif in western Alps, Italy. The sample is massive and fine-grained containing glaucophane (~55%), epidote

(~20%), plagioclase (~10%), garnet (~5%), titanite (~5%), and chlorite (~5%). The composition of glaucophane was measured using the JEOL JXA-8100 electron probe X-ray microanalyzer at the Center for Research Facilities at Gyeongsang National University, Korea. The measurement conditions included accelerating voltage of 15 kV, current of 10 nA, and beam size of 5 x 5 μm^2. The derived chemical formula of glaucophane was $Na_{2.0}[(Mg_{1.7}Fe_{1.3})(Ca_{0.04}Mn_{0.01})Al_{1.8}Fe_{0.09}]Si_{8.1}O_{22}(OH)_2$. Using a modified Griggs apparatus[14] housed at the Tectonophysics Laboratory at the School of Earth and Environmental Sciences (SEES) at Seoul National University (SNU), a core-drilled blueschist sample with 3 mm diameter was pressurized up to 2 GPa over 12 hours and then heated up to 730 ºC using a graphite heater in 30 minutes, which was held for 9 hours. The experiments were performed total seven times at different P-T conditions (Supplementary Table 2). The temperature was monitored using a B-type thermocouple to the maximum uncertainties of ±10 ºC, as indicated by temperature differences of the two thermocouples, while the uncertainties in pressure was estimated to be less than ~5%[15]. After the P-T run, the sample was quenched to room temperature. Field Emission-Scanning Electron Microscopy with Energy Dispersive Spectroscopy (FE-SEM-EDS) measurement was carried out on the recovered core sample using the FE-SEM (JSM 7100F) and the EDS system (Oxford Instruments) at the SEES at SNU. Back-scattered electron image of the recovered sample was also obtained using the same FE-SEM (JSM 7100F), which was operated at an accelerating voltage of 15 kV and a working distance of 10 mm.

7. A Paris-Edinburgh Cell experiment on a mixture of glaucophane, jadeite, and talc

In-situ high-pressure and high-temperature synchrotron energy dispersive X-ray powder diffraction experiments using a Paris-Edinburgh Cell[16] were conducted at beamline 16-BM-B of HPCAT at the APS. The starting material was a mixture of glaucophane, jadeite, and talc in an 1:2:1 molar ratio for the equilibrium reaction. Pressure were determined by X-ray diffraction of MgO[16]. The sample was heated with a graphite cylinder, with temperatures estimated from a previously calibrated power-temperature curve with an identical assembly[17]. The uncertainties in pressure and temperature are about 0.3 GPa and 100 K, respectively[16,17]. The experimental target P-T conditions were up to 3.1 GPa and 1000 ºC which was held for 4 hours to observe the breakdown of glaucophane and down to 2.1 GPa and 550 ºC which was held for 6 hours to observe the regrowth of glaucophane peaks.

(a)

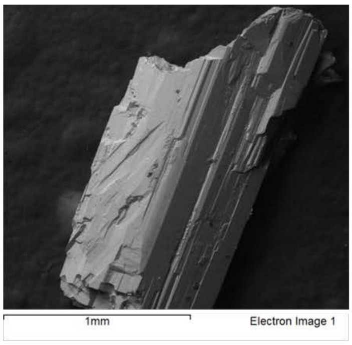

Oxide form	(%)
SiO_2	60.15
Al_2O_3	10.46
FeO	6.91
MgO	13.68
Na_2O	8.15
CaO	0.65
Total	100.00

T	C	B
8.0	5.1	2.2

(b)

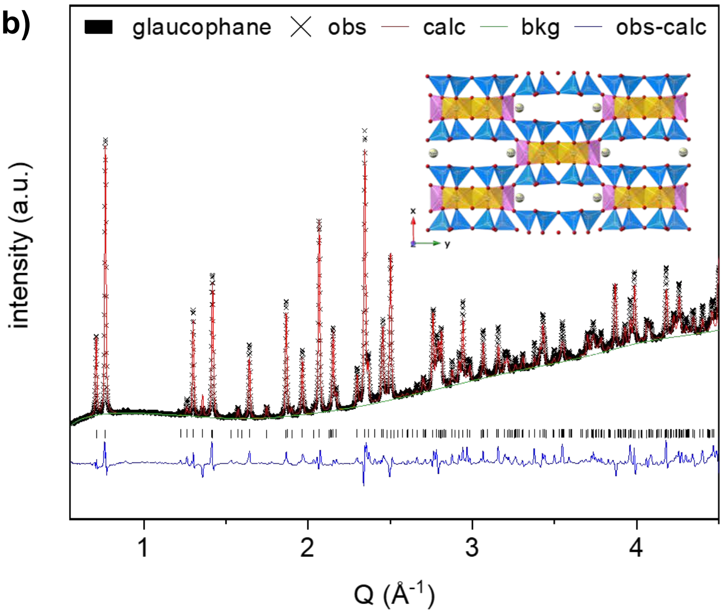

* Amphibole group: $A_{0\text{-}1}B_2C_5T_8O_{22}(OH)_2$

* Structural formula is calculated from chemical compositions, based on 23 oxygen atoms

* Chemical formula (EDS): $Na_{2.1}Ca_{0.1}(Mg_{2.7}Fe_{0.8}Al_{1.6})Si_{8.0}O_{22}(OH)_2$

* Chemical formula (Rietveld refinement): $Na_{1.9}Ca_{0.1}(Mg_{2.7}Fe_{0.5}Al_{1.8})Si_8O_{22}(OH)_2$

Supplementary Figure 1. Chemical and crystallographic properties of glaucophane. (a) SEM image of a glaucophane crystal with its chemical composition as derived from the energy dispersive X-ray spectroscopy. (b) The Rietveld fit of the X-ray diffraction pattern of glaucophane measured at ambient conditions. A polyhedral representation of the refined crystal structure of glaucophane is shown in the inset, and the refined chemical formula is compared to that from the EDS analysis in the bottom.

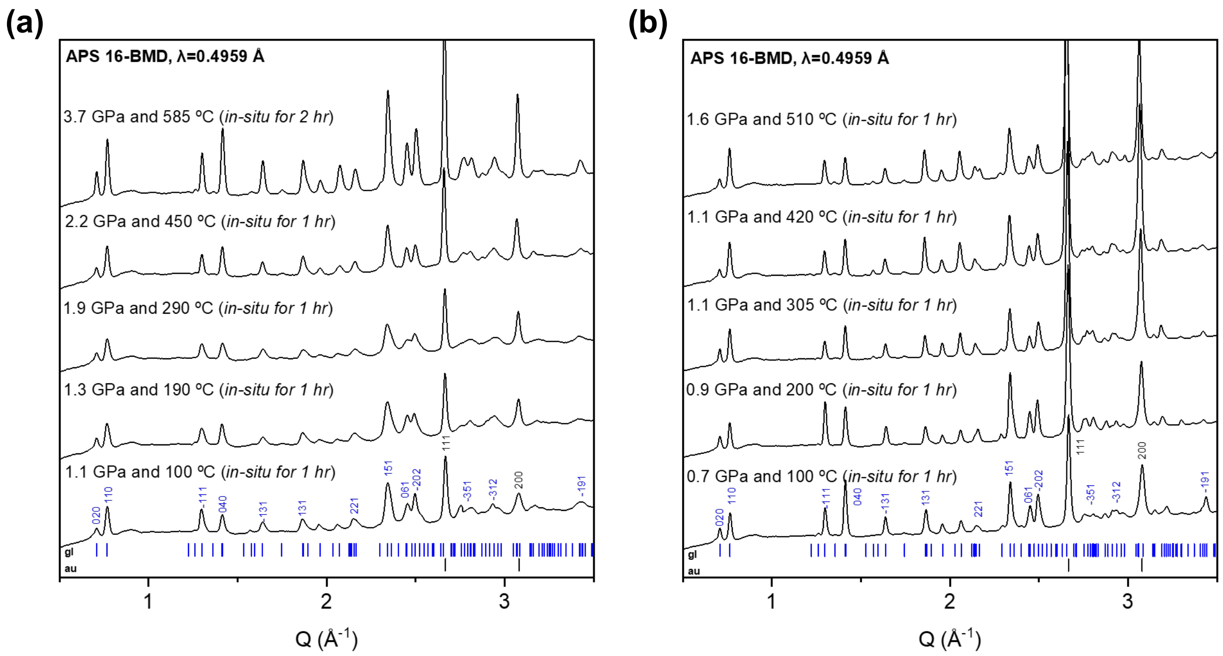

Supplementary Figure 2. X-ray diffraction patterns of glaucophane under the P-T conditions of the cold and warm subduction geotherms. X-ray powder diffraction patterns were measured after subjecting glaucophane along the P-T conditions of (a) cold and (b) warm subduction zones for 1 or 2 hours under 4 wt.% H_2O medium before respective breakdowns at higher P-T conditions. Phase abbreviations: glaucophane (gl) and gold (au).

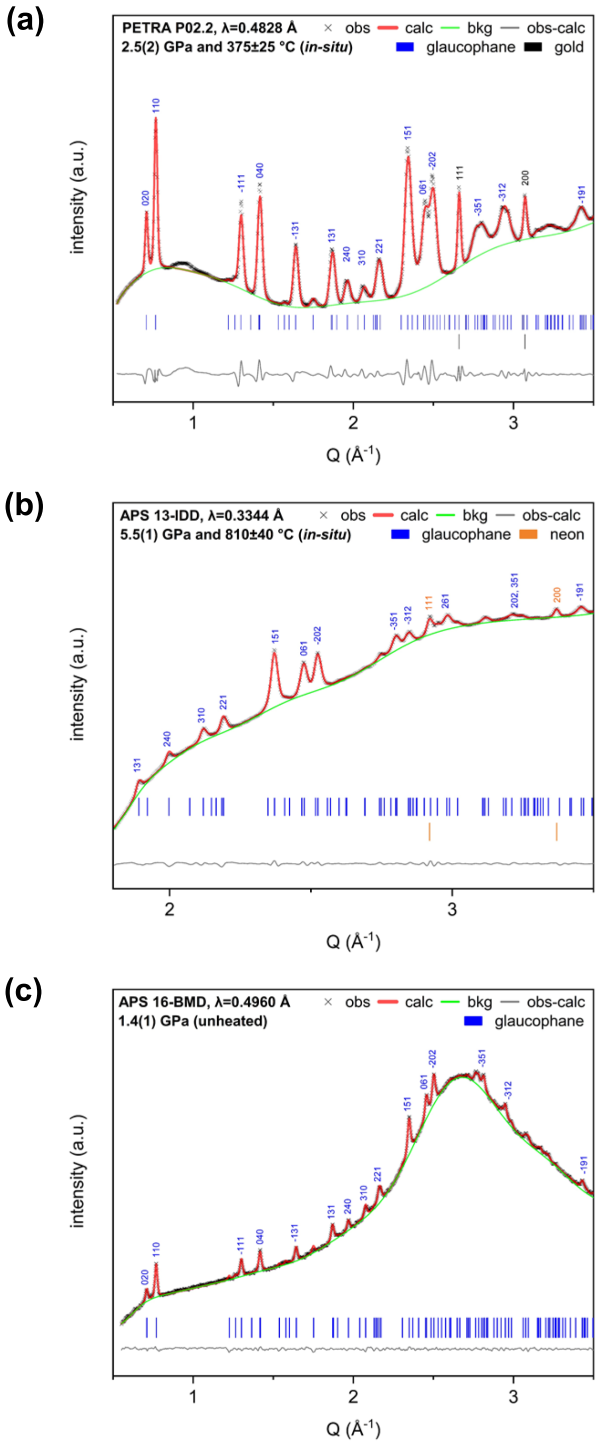

Supplementary Figure 3. The representative profile fits of the original XRD patterns of glaucophane measured at different synchrotron beamlines. (a) PETRA P02.2 beamline, (b) APS 13-IDD beamline, and (c) APS 16-BMD beamline. The experimental data are shown in black crosses while the profile fits using the LeBail method are shown in red lines. Note the differences in the backgrounds shown in green lines due to the differences in the DAC, X-ray energy, type of detectors used, and the sample-to-detector settings, etc. (see Methods for more details).

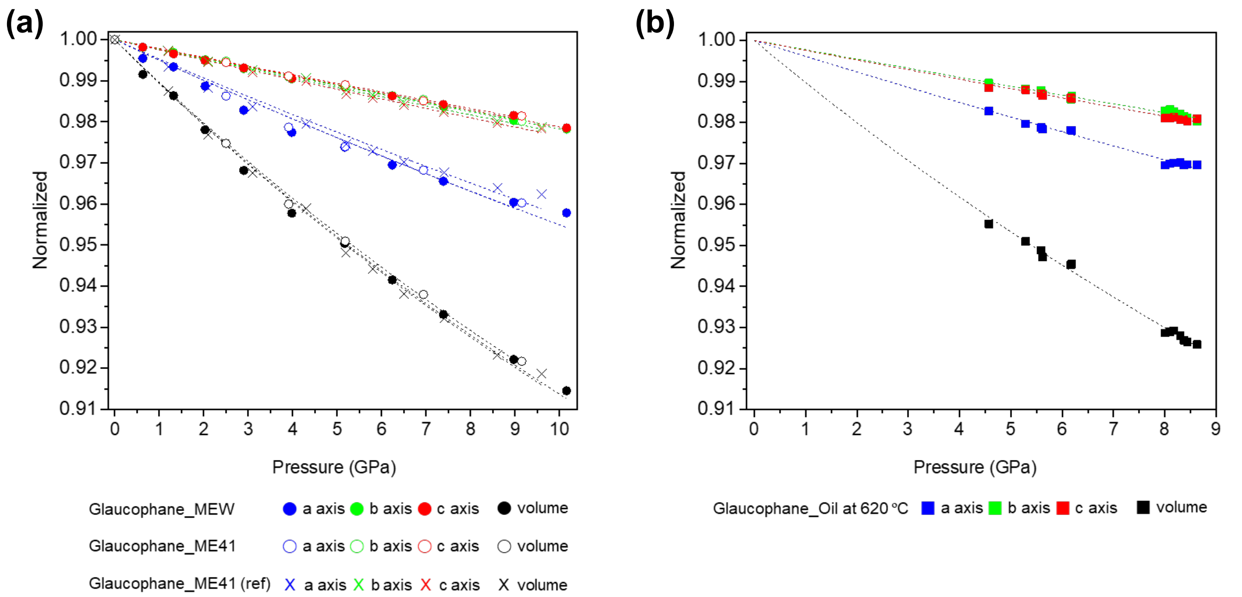

Pressure Medium	β_a ($kbar^{-1}$)	β_b ($kbar^{-1}$)	β_c ($kbar^{-1}$)	K_0 (GPa)
MEW	$5.1(1) \times 10^{-4}$	$2.3(1) \times 10^{-4}$	$2.2(1) \times 10^{-4}$	92(1)
ME41	$5.1(1) \times 10^{-4}$	$2.3(1) \times 10^{-4}$	$2.2(1) \times 10^{-4}$	94 (1)
ME41(ref)	$4.8(1) \times 10^{-4}$	$2.4(1) \times 10^{-4}$	$2.5(1) \times 10^{-4}$	91 (1)

Pressure Medium	β_a ($kbar^{-1}$)	β_b ($kbar^{-1}$)	β_c ($kbar^{-1}$)	K_0 (GPa)
Oil	$3.9(1) \times 10^{-4}$	$2.3(1) \times 10^{-4}$	$2.4(1) \times 10^{-4}$	95(1)

Supplementary Figure 4. Compression behavior of glaucophane at room temperature and high temperature. The linear compressibilities (β in $kbar^{-1}$) and bulk modulus (K in GPa) of glaucophane (a) under methanol:ethanol:water = 16:3:1 (MEW) and methanol:ethanol = 4:1 (ME41) medium at ambient temperature, compared to those (b) under silicone oil medium at 620±10 ºC. The results from previous study at ambient condition are shown for comparison[18]. *The standard deviations for the experimental fits are given in parentheses. The parameters are derived by least-squares regression, using EOSFit[19], of the observed data to the Murnaghan equation of state[20]: $P_{VT} = K_{0T}/K'_{0T}[(V_{0T}/V)^\wedge(K'_{0T})\text{-}1]$.

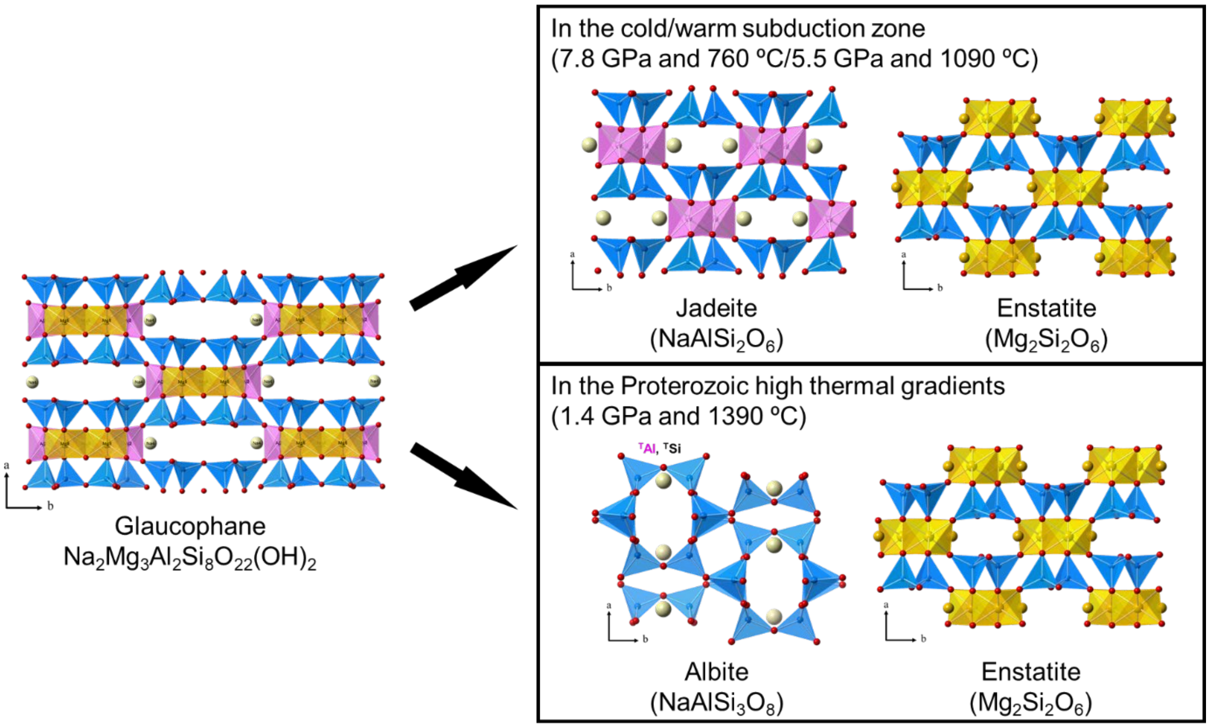

**Cold/Warm subduction zones**
1) 7.8(3) GPa and 760±45 ºC/ 5.5(1) GPa and 1090±50 ºC<br>Glaucophane = 2 Jadeite + 1.5 Enstatite + Coesite + Fluid<br>$Na_2Mg_3Al_2Si_8O_{22}(OH)_2 = 2\ NaAlSi_2O_6 + 1.5\ Mg_2Si_2O_6 + SiO_2 + H_2O$
**Subduction zone model in the Proterozoic tectonic setting**
2) 1.4(1) GPa and 1390±30 ºC<br>Glaucophane → Albite + Enstatite + Fluid<br>$Na_2Mg_3Al_2Si_8O_{22}(OH)_2 \rightarrow NaAlSi_3O_8 + Mg_2Si_2O_6 + H_2O$

Supplementary Figure 5. Structural and chemical changes of glaucophane. Glaucophane decomposes in different ways in cold/warm subduction conditions and in the Proterozoic high thermal gradients.

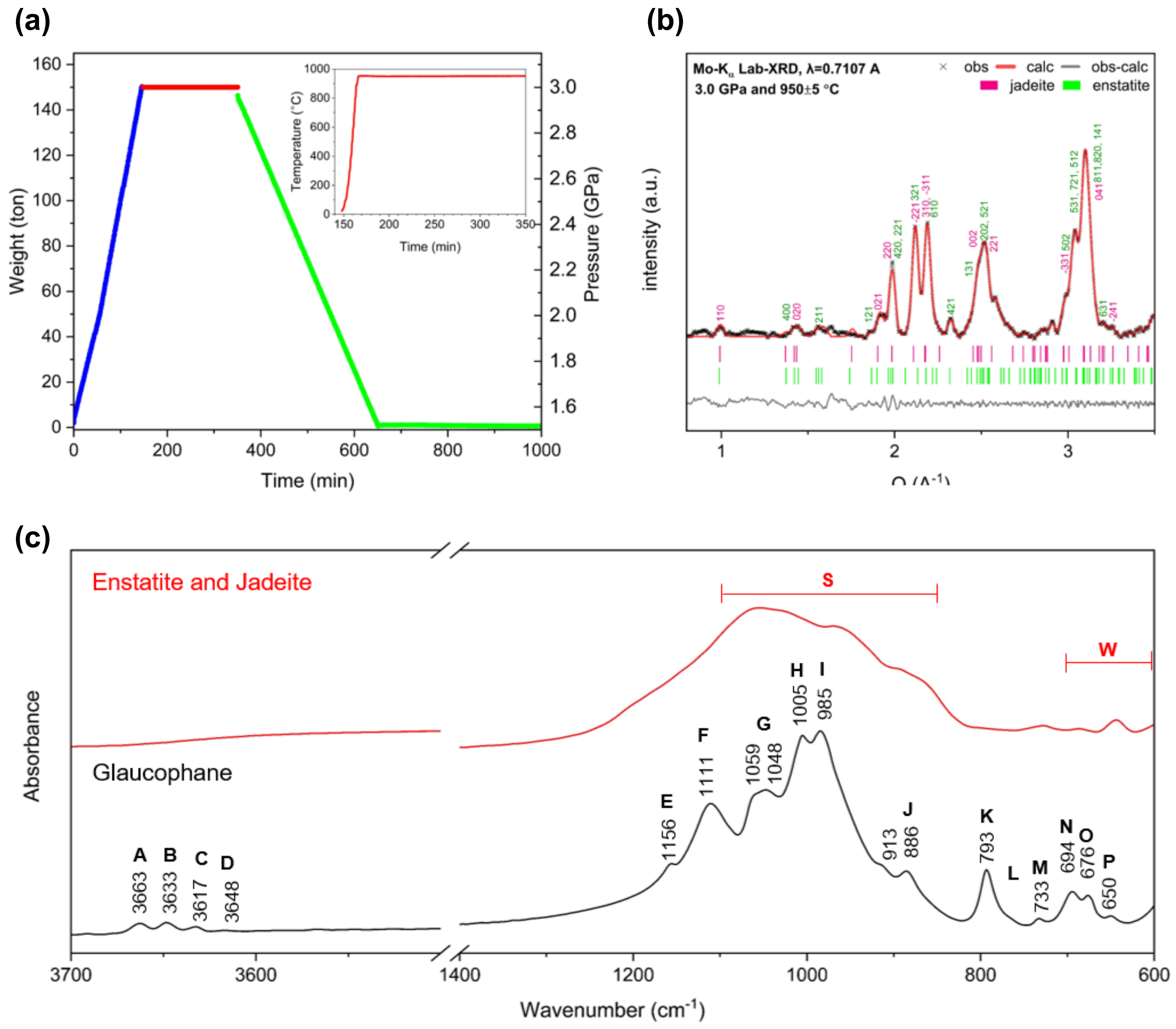

Supplementary Figure 6. Results from the multi-anvil press experiment on glaucophane. (a) P-T cycle during the multi-anvil press experiment. (b) The profile fit of the experimental X-ray powder diffraction pattern of the recovered sample measured using Rigaku MicroMax-007HF XRD with Mo target (λ = 0.7107 Å). The experimental data are shown in black crosses while the fit using the LeBail method is shown in red line. (c) FT-IR spectra of the original and the recovered sample. The spectra of the original glaucophane (black line) exhibit four hydroxyl bands in the region of 3600-3700 cm^{-1} (A-D) and twelve lattice vibrational bands in the region of 600-1300 cm^{-1} (E-P)[21,22] (Supplementary Table 3). In contrast, the spectra of the recovered sample (red line) show the disappearance of the O-H stretching modes and spectroscopic features of pyroxene structures with strong infrared absorption bands in the region of 850-1100 cm^{-1} (stretching motions of Si-O) and weak bands in the region of 600-700 cm^{-1} (vibrations of Si-O-Si linkages) (S-W), which are associated to the single chain of silica tetrahedra and the chains bound together by magnesium octahedra[23] (Supplementary Table 3). These results confirm the completion of glaucophane dehydration to pyroxenes (enstatite and jadeite) at 3 GPa and 950±5 ºC.

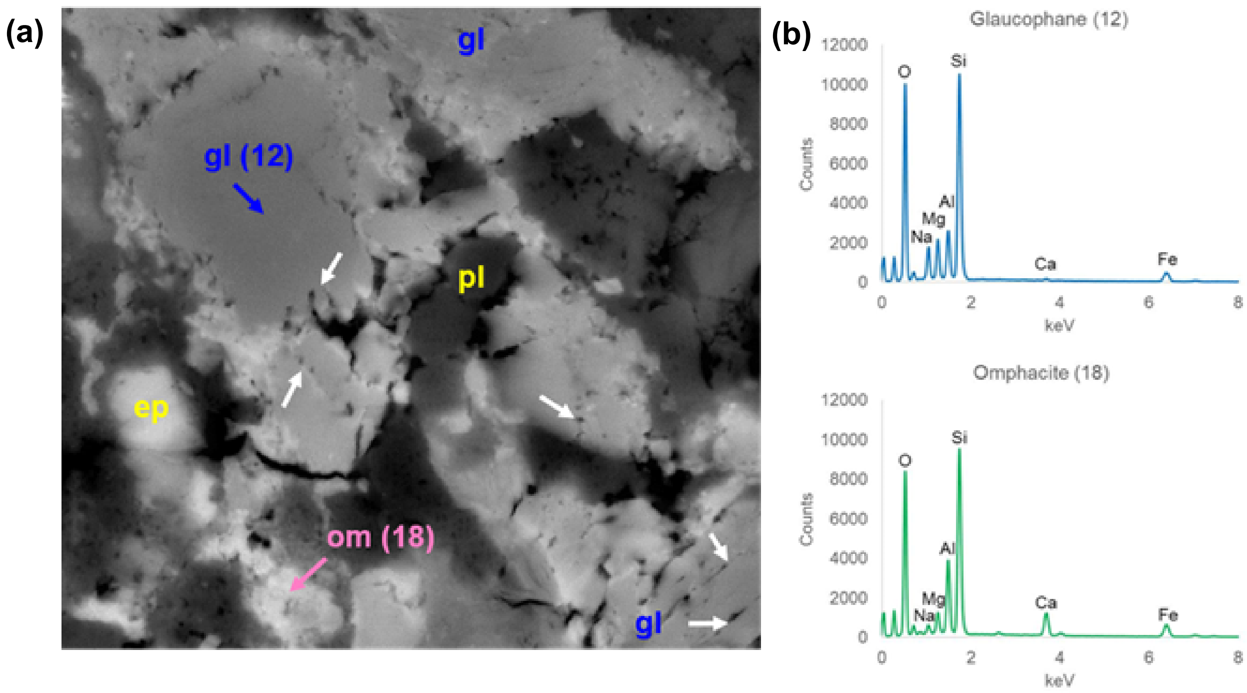

Supplementary Figure 7. A modified Griggs apparatus experiment of natural epidote blueschist rock showing dehydration features of glaucophane from 2 GPa and 670±10 ºC conditions. (a) Back-scattered electron (BSE) image showing partially dehydrated glaucophane (gl) with rugged grain boundaries and fluid inclusion trails (white arrows); consequently, omphacite (om) has formed as the dehydration product. Phase abbreviations: epidote (ep) and plagioclase (pl). (b) Energy Dispersive Spectroscopy spectra of glaucophane (12) and omphacite (18) as labeled in the BSE image.

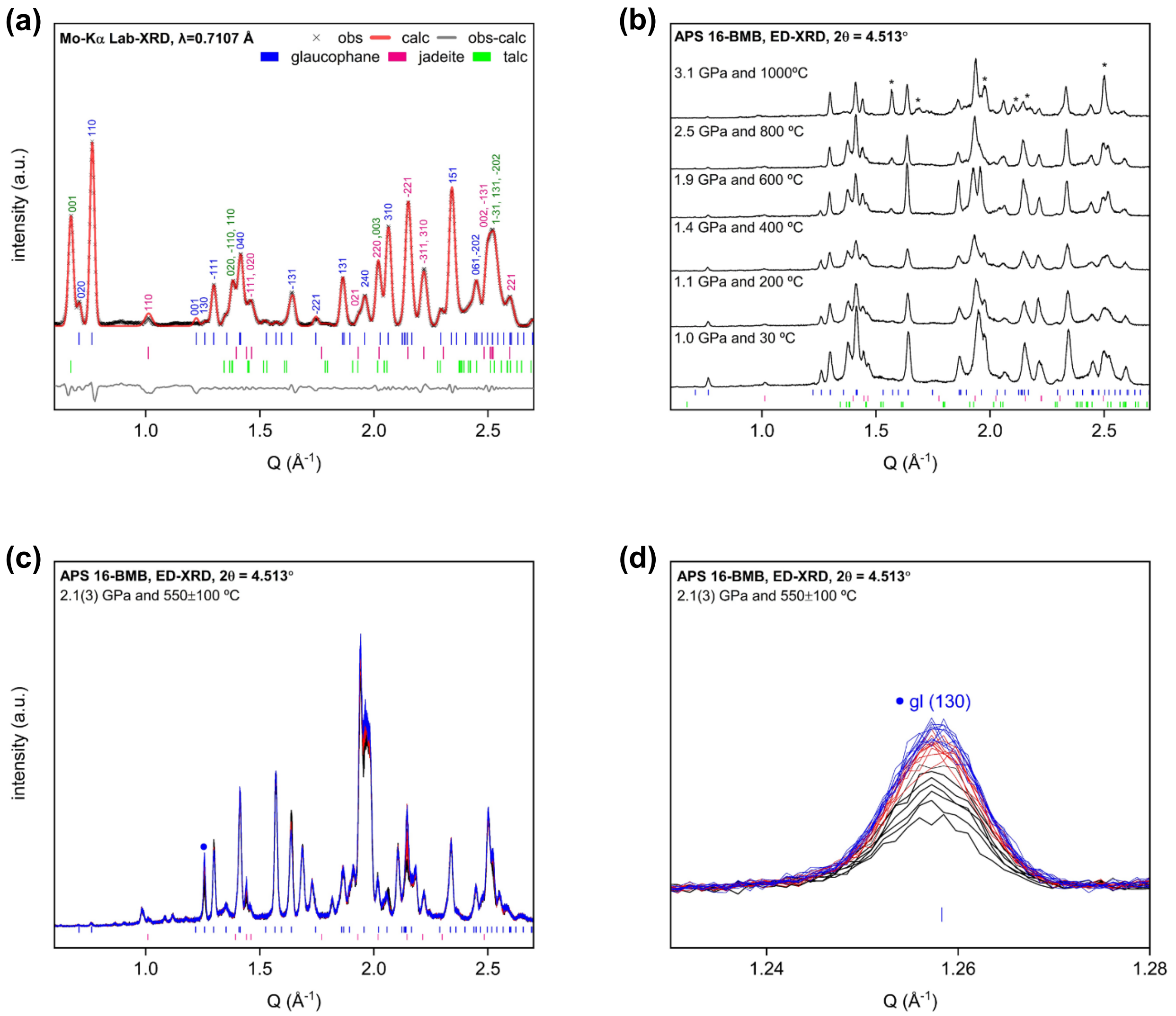

Supplementary Figure 8. Reversal experiments using a Paris-Edinburgh Cell. (a) The profile fit of the experimental X-ray powder diffraction pattern of a mixture of glaucophane, jadeite, and talc measured using Rigaku MicroMax-007HF XRD with Mo target (λ = 0.7107 Å). The experimental data are shown in black crosses while the fit using the LeBail method is shown in red line. (b) A stacked plot of energy-dispersive X-ray powder diffraction (ED-XRD) patterns on the mixture of glaucophane, jadeite, and talc heated up to 1000±100 ºC at 3.1(3) GPa. (c) in-situ ED-XRD patterns on the mixture measured over 6 hours after reducing the temperature to 550±100 ºC at 2.1(3) GPa. (d*)* the growth of glaucophane (130) peak (blue dot in c) over 6 hours at 550 ºC and 2.1 GPa (black lines – early time, red lines – after 3 hours, blue lines – after 5 hours).

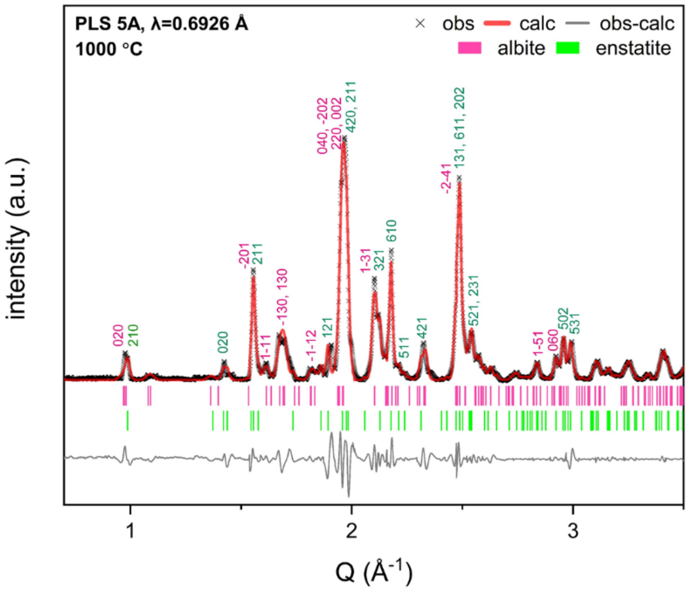

Supplementary Figure 9. Decomposition of glaucophane at ambient pressure. The profile fit of the X-ray powder diffraction pattern of glaucophane after quenching from heating at 1000 ºC for one hour. The experimental data are shown in black crosses while the fit using the LeBail method is shown in red line. Glaucophane decomposes into albite (ab) and enstatite (en) at high temperature at ambient pressure.

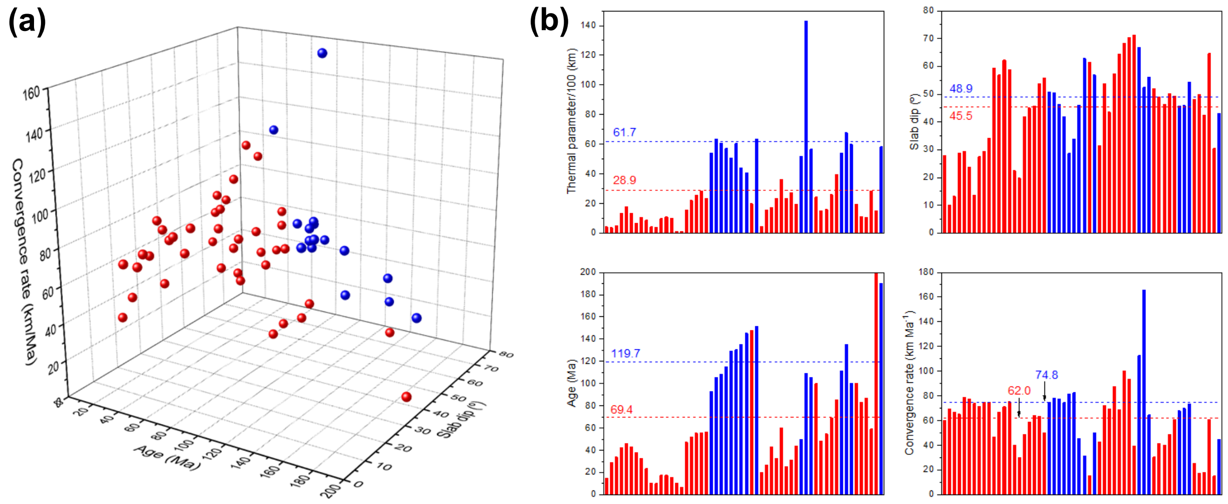

Supplementary Figure 10. Plots of 56 subduction system (a) A 3D scatter and (b) bar plots of slap dip, age, convergence rate, and thermal parameter of 56 subduction zones from Syracuse et al. (2010)[6] (Supplementary Table 5). Grouping into warm and cold subduction zones is marked in red and blue, respectively, with the corresponding average values of slap dip, age, convergence rate, and thermal parameter.

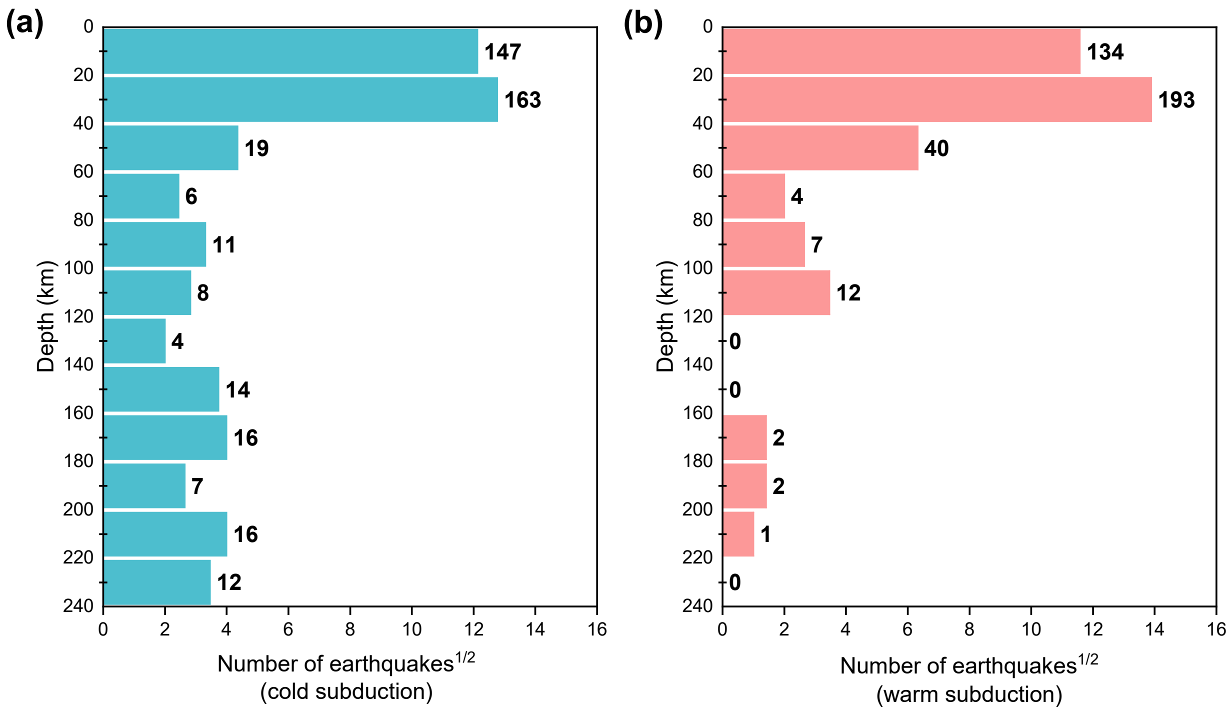

Supplementary Figure 11. The frequency of the earthquake occurrence along the Tonga trench and Chile trench over the past 10 years (ISC-EHB Bulletin, investigation period between Jan. 2006 ~ Jan.2016)[24-26]. The number of the earthquake occurrence is grouped in every 20 km depths down to 240 km along (a) the Tonga trench and (b) the Chile trench.

Supplementary Table 1. Crystal chemical data and crystallographic details pertaining to the structure refinement of glaucophane including atomic positions, occupancy, and displacement parameters at ambient condition.

Glaucophane (Ambient)			
Space group			C2/m
R_{wp} (%), χ^2			2.16, 1.09
Chemical composition	(EDS)		$Na_{2.1}Ca_{0.1}(Mg_{2.7}Fe_{0.8}Al_{1.6})Si_8O_{22}(OH)_2$
	(Rietveld)		$Na_{1.9}Ca_{0.1}(Mg_{2.7}Fe_{0.5}Al_{1.8})Si_8O_{22}(OH)_2$
Cell Parameter	a	(Å)	9.5271(3)
	b	(Å)	17.7085(6)
	c	(Å)	5.2867(2)
	β	(°)	103.683(2)
Volume	V	($Å^3$)	866.6(1)

atom	x	y	z	Occupancy	U_{iso}
Mg1	0.0000	0.0892(2)	0.5000	0.97(1)	0.009(6)
Fe1	0.0000	0.0892(2)	0.5000	0.07(1)	0.009(6)
Al2	0.0000	0.1805(1)	0.0000	0.89(1)	0.004(6)
Fe2	0.0000	0.1805(1)	0.0000	0.09(1)	0.004(6)
Mg3	0.0000	0.0000	0.0000	0.80(1)	0.005(7)
Fe3	0.0000	0.0000	0.0000	0.20(1)	0.005(7)
Na4	0.0000	0.2766(5)	0.5000	0.94(1)	0.014(6)
Ca4	0.0000	0.2766(5)	0.5000	0.04(1)	0.014(6)
Si1	0.2871(2)	0.0876(1)	0.2941(2)	1	0.002(1)
Si2	0.2909(1)	0.1727(1)	0.8037(2)	1	0.002(1)
O1	0.1121(2)	0.0921(1)	0.2082(2)	1	0.001(1)
O2	0.1152(1)	0.1717(1)	0.7414(2)	1	0.001(1)
O4	0.3535(3)	0.2583(3)	0.8057(2)	1	0.001(1)
O5	0.3550(1)	0.1343(1)	0.0871(2)	1	0.001(1)
O6	0.3435(1)	0.1238(1)	0.5823(3)	1	0.001(1)
O7	0.3378(3)	0.0000	0.2986(3)	1	0.001(1)
O3H	0.1130(1)	0.0000	0.7082(5)	1	0.001(1)

* The e.s.d. values are in parentheses. Isotropic displacement factors (U_{iso}) were refined by grouping the framework tetrahedral atoms and the framework oxygen atoms. Restraints on the T-O, M1-O, M2-O M3-O, and O-O bond distances have been applied.

Supplementary Table 2. Experimental P-T conditions simulating diverse subduction environments. P-T conditions for cold and warm subduction zones are further divided into slab surface and slab Moho (beneath ~7 km depth of oceanic crust). P-T conditions for the Proterozoic tectonic settings with high thermal gradients are included along with atmospheric pressure conditions as reference.

cold subduction (in-situ & ex-situ)									warm subduction (in-situ & ex-situ)						Proterozoic (ex-situ)			Atmosphere (ex-situ)		
slab surface			between surface and Moho			slab Moho			slab surface			slab Moho								
P (GPa)	T (ºC)	T (min)	P (GPa)	T (ºC)	T (min)	P (GPa)	T (ºC)	T (min)	P (GPa)	T (ºC)	T (min)	P (GPa)	T (ºC)	T (min)	P (GPa)	T (ºC)	T (min)	P (GPa)	T (ºC)	T (min)
0.50	23	-	1.29	190	15	0.61	50	30	0.7	100	60	2.0*	500	540	1.42	1245	3	0	800	60
1.05	100	60	1.83	235	20	1.10	70	30	0.9	200	60	2.85	505	5	1.42	1095	3	0	900	60
1.1	100	60	2.36	290	15	1.14	80	30	0.7	260	120	2.85	645	5	1.42	1300	3	0	1000	60
1.25	90	90	2.32	335	40	1.58	145	60	1.1‡	200	20	2.85	725	5	1.42	1390	3			
1.57	125	10	2.46	375	35	2.18	200	30	1.1	305	60	5.48	810	2						
1.92	180	10	2.61	535	30	2.68	225	60	1.0	360	120	5.48	810	2						
1.3	190	60	3.04	570	15	3.97	350	30	1.4‡	400	20									
1.39	280	120	3.20	580	60	4.44	400	30	1.1	420	60									
1.9	290	60	3.32	575	10	5.61	450	10	0.9*	430	540									
2.42	300	60	3.43	575	10				1.2*	480	540									
2.26	390	20	3.53	575	20				1.5*	500	540									
2.46	435	10	3.84	575	20				1.6	510	60									
2.2	450	60	4.10	570	15				1.9	560	120									
2.62	460	25	5.29	580	20				1.9‡	600	40									
2.66	500	20	5.07	585	20				2.1‡	550	360									
3.26	500	60	5.28	580	15				2.0*	570	540									
2.93	550	60	5.84	580	40				2.0*	670	540									
3.7	585	120	6.35	580	20				2.0*	730	540									
3.35	670	40	7.11	615	10				2.5‡	800	40									
3.69	725	40	7.58	660	25				3.00†	950	240									
3.69	745	15	7.82	760	20				3.1‡	1000	240									
									5.48	955	2									
									5.48	955	2									
									5.48	1030	2									
									5.48	1055	2									
									5.48	1090	2									

abbreviations: Pressure (P), Temperature (T), and Time (t)

* Modified-Griggs apparatus † Multi-anvil press ‡ Paris-Edinburgh Cell

Supplementary Table 3. Assignments of the measured IR bands. IR spectra measured from the original and the recovered products of glaucophane from the multi-anvil press experiment (Supplementary Fig. 6) are assigned for various stretching and vibration modes.

Name	Measured frequencies	Vibrational assignment
A	3663	O-H (stretching)
B	3648	O-H (stretching)
C	3633	O-H (stretching)
D	3617	O-H (stretching)
E	1156	ν_{as} Si-O-Si, O-Si-O (stretching)
F	1111	ν_{as} Si-O-Si, O-Si-O (stretching)
G	1059, 1048	ν_{as} Si-O-Si, O-Si-O (stretching)
H	1005	ν_{as} Si-O-Si, O-Si-O (stretching)
I	985	ν_{as} Si-O-Si, O-Si-O (stretching)
J	913, 886	ν_s Si-O-Si (stretching)
K	793	ν_s Si-O-Si, Si-O (stretching)
M	733	ν_s Si-O-Si, Si-O (stretching)
N	694	O-H (libration)
O	676	O-H (libration), lattice (vibration)
P	650	ν_s Si-O-Si, Si-O (stretching)

Name	Measured frequencies region	Vibrational assignment
S	850-1100	Si-O (stretching)
W	600-700	Si-O-Si linkage (vibration)

Supplementary Table 4. Estimation of the global H_2O influx. Global H_2O influx has been estimated based on the amount of H_2O in amphiboles in the oceanic crust.

**Parameters**	**value**	**Reference**
thickness of oceanic crust[27,28] (T)	5-8 km (av. 7 km)	White et al. (1992)<br>Geissler et al. (2017)
surface area of oceanic crust[29] (A)	309 x 10^6 km^2	Fowler (2005)
volume of oceanic crust[30] (V)	2100 x 10^6 km^3	Wyllie (1971)
density of oceanic crust[31] (ρ)	2.86 Mg m^{-3} (2.86x10^6 g m^{-3})	Carlson and Herrick (1990)
mass of oceanic crust[32] (M)	6.07 x 10^{21} kg	Ronov and Yaoshevsky (1969)
mean depth of ocean[33] (t)	av. 3.7 km	Charette and Smith (2010)
surface area of ocean[33] (a)	361 x 10^6 km^2	Charette and Smith (2010)
volume of ocean[33] (v)	1332 x 10^6 km^3	Charette and Smith (2010)
mass of ocean[34] (m)<br>ex) Arctic Ocean	1.4 x 10^{21} kg<br>0.02 x 10^{21} kg	Mackenzie (1966)
basalt wt.% in oceanic crust[32]	88.8 wt.% → 5.39 x 10^{21} kg	Ronov and Yaoshevsky (1969)
wt.% H_2O in oceanic crust[35]	5-6 wt.% H_2O	Schmidt and Poli (1998)
wt.% of amphiboles in oceanic crust[35]	20-60 wt.% of a basalt	Schmidt and Poli (1998)
mass of amphiboles in oceanic crust	1.08-3.23 x 10^{21} kg	calculated data from<br>Ronov and Yaoshevsky (1969)<br>Schmidt and Poli (1998)
wt.% H_2O in amphibole[36]	2.2-2.3 wt.% H_2O	Faccenda (2014), this study
amount of H_2O due to dehydration of amphibole	2.38-7.43 x 10^{19} kg<br>1.7-5.3 % of ocean	Estimated from this study
amount of released H_2O/volume of oceanic basalts	1.1-3.5 x 10^4 g H_2O m^{-3}	Estimated from this study
released wt.% H_2O from amphibole in the oceanic crust	0.39-1.22 wt.% H_2O<br>7-20 % of H_2O in oceanic crust	Estimated from this study
amount of H_2O injected to the deep mantle through amphibole in the cold subduction zone	0.7-2.1 x 10^{19} kg<br>0.5-1.5 % of ocean (ca. Arctic Ocean)	Estimated from this study
H_2O flux into subduction zones[37]	0.7-1.0 x 10^{12} kg yr^{-1}	van Keken et al. (2011)
H_2O flux in the mantle[37]	0.22-0.34 x 10^{12} kg yr^{-1}	van Keken et al. (2011)
H_2O flux to the mantle through amphibole in the cold subduction zone	0.03-0.11 x 10^{12} kg yr^{-1}<br>ca. 4-11 % of H_2O flux into subduction zones	Estimated from this study<br>(assumed that cycle of oceanic crust is 200 Ma)

Supplementary Table 5. Grouping of warm and cold subduction system. The 56 subduction zones from Syracuse et al. (2010)[6] are grouped into warm and cold subduction zones based on the differences in the slap dip, age, convergence rate, and thermal parameter.

	Cold subduction zone name	Slag dip (º)	Age (Ma)	Convergence rate (km Ma^{-1})	Thermal parameter/100 (km)
1	Kamchatka	50.7	92.9	75.1	54.1
2	North Kurile	50.4	105.6	78.3	63.6
3	South Kurile	46.4	108.5	77.4	60.7
4	Hokkaido	41.9	115.2	74.7	57.2
5	North Honshu	28.7	129.3	81.6	50.7
6	Central Honshu	33.9	130.5	82.7	60.4
7	Izu	46.1	135.4	45.6	44.1
8	Bonin	62.8	145.3	31.6	40.8
9	South Marianas	56.9	151.6	50	63.5
10	South Vanuatu	66.8	50.0	112.7	51.8
11	Tonga	52.4	109.0	165.8	143.2
12	Kermadec	56.1	105.6	64.6	56.6
13	Java	45.8	111.2	67.8	54.0
14	Bali/Lombok	45.9	134.9	69.8	67.7
15	West Banda Sea	54.4	100.0	73.6	59.8
16	calabria	43.0	190.0	45	58.3
	Average	48.9	119.7	74.8	61.7

	Warm subduction zone name	Slag dip (º)	Age (Ma)	Convergence rate (km Ma^{-1})	Thermal parameter/100 (km)
1	Colombia/Ecuador	28	15	60	4.2
2	Northern Peru Gap	10.2	29	69.5	3.6
3	Central Peru Gap	13.2	33.9	66.7	5.2
4	Peru	28.7	42.1	65.1	13.4
5	North Chile	29.3	46.2	79	17.8
6	North-Central Chile	23.6	42.8	77.4	13.3
7	Central Chile Gap	13.7	37.7	73.8	6.6
8	Central Chile	27.5	32.4	71.6	10.7
9	South-Central Chile	29.5	23.5	74.7	8.6
10	South Chile	34.2	10.3	74.9	4.3
11	Mexico	59.5	10	47	4
12	Guatemala/El Salvador	56.9	17.4	66.9	9.7
13	Nicaragua	62.2	17.5	71.1	11
14	Costa Rica	58.9	15.8	75.4	10.1
15	North Cascadia	22.4	10.6	40	1.3
16	Central Cascadia	19.8	6.9	30	1
17	Alaska	42	47.1	49	15.6
18	Alaska Peninsula	45	52.2	59	22
19	East Aleutians	45.6	55.3	64.2	25.4
20	Central Aleutians	53.7	55.9	63.4	28.4
21	West Aleut	55.9	56.1	50.2	23.5
22	North Marianas	61.5	147.8	15.3	19.9
23	Nankai	31.4	20	43	4.5
24	Kyushu	53.8	27	72	15.6
25	Ryukyu	43.5	43	69.5	17.1
26	North Philippines	57.4	32.4	87.7	23.6
27	South Philippines	64.3	59.8	69	36.3
28	New Britain	68.2	25	100.4	23.1
29	Solomon	70.4	31	93.6	27.1
30	North Vanuatu	71.3	44	39.6	19.4
31	New Zealand	52	100	30.4	24.2
32	North Sumatra	49.1	48.3	41.5	15.1
33	Central Sumatra	46.4	54.8	40	15.9
34	South Sumatra	50.2	68.9	48.7	25.9
35	Sunda Strait	49.3	85.5	61	39.6
36	East Banda Sea	48	100	25.3	19.5
37	North Lesser Antilles	49.8	83	17.6	11.1
38	South Lesser Antilles	42.4	86.6	17.9	10.4
39	Scotia	64.6	59.1	60.8	28.6
40	Aegean	30.5	200	15	15.2
	Average	44.1	49.3	56.9	15.8

* The thermal parameter (ϕ) of a slab is defined as the product of the slab age (a), convergence rate (v_c), and the sin of the slab dip angle ($sin(\alpha)$): $\phi = av_c sin(\alpha)$[38,39] and is used to quantify the thermal state of a subducting slabs[40,41].

Supplementary References

1. Hammersley, A. P. FIT2D: An introduction and Overview: ESRF Internal Report, ESRF97HA02T; 1997.

2. Toby, B. H. EXPGUI, a graphical user interface for GSAS. *J. Appl. Crystallogr.* **34**, 210-213 (2001).

3. Larson, A. C. & Von Dreele, R. B. GSAS-General Structure Analysis Sytem (Los Alamos National Laboratory, 1986).

4. Lorenzana, H., Bennahmias, M., Radousky, H. & Kruger, M. Producing diamond anvil cell gaskets for ultrahigh-pressure applications using an inexpensive electric discharge machine. *Rev. Sci. Instrum.* **65**, 3540-3543 (1994).

5. Mao, H. K., Xu, J. & Bell, P. M. Calibration of the ruby pressure gauge to 800 kbar under quasi-hydrostatic conditions. *J. Geophys. Res. Solid Earth* **91**, 4673-4676 (1986).

6. Syracuse, E. M., van Keken, P. E. & Abers, G. A. The global range of subduction zone thermal models. *Phys. Earth Planet. Inter.* **183**, 73-90 (2010).

7. Wang, Y. et al. Thermal equation of state of copper studied by high P-T synchrotron x-ray diffraction. *Appl. Phys. Lett.* **94**, 071904 (2009).

8. Liermann, H.-P. et al. Experimental method for in situ determination of material textures at simultaneous high pressure and high temperature by means of radial diffraction in the diamond anvil cell. *Rev. Sci. Instrum.* **80**, 104501 (2009).

9. Liermann, H.-P. et al. The extreme conditions beamline P02.2 and the extreme conditions science infrastructure at PETRA III. *Journal of synchrotron radiation* **22**, 908-924 (2015).

10. Prakapenka, V. B. et al. Advanced flat top laser heating system for high pressure research at GSECARS: application to the melting behavior of germanium. *High. Pressure. Res* **28**, 225-235 (2008).

11. Prescher, C. & Prakapenka, V. B. DIOPTAS: a program for reduction of two-dimensional X-ray diffraction data and data exploration. *High. Pressure. Res* **35**, 223-230 (2015).

12. Boehler, R. Laser heating in the diamond cell: techniques and applications. *Hyperfine Interact.* **128**, 307-321 (2000).

13. Kunimoto, T., Irifune, T. & Sumiya, H. Pressure generation in a 6-8-2 type multi-anvil system:

a performance test for third-stage anvils with various diamonds. *High. Pressure. Res* **28**, 237-244 (2008).

14. Jung, H. Crystal preferred orientations of olivine, orthopyroxene, serpentine, chlorite, and amphibole, and implications for seismic anisotropy in subduction zones: a review. *Geosci. J* **21**, 985-1011 (2017).

15. Jung, H. & Green, H. W. Experimental Faulting of Serpentinite during Dehydration: Implications for Earthquakes, Seismic Low-Velocity Zones, and Anomalous Hypocenter Distributions in Subduction Zones. *International Geology Review* **46**, 1089-1102 (2004).

16. Kono, Y., Irifune, T., Higo, Y., Inoue, T. & Barnhoorn, A. P-V-T relation of MgO derived by simultaneous elastic wave velocity and in situ X-ray measurements: A new pressure scale for the mantle transition region. *Phys. Earth Planet. Inter.* **183**, 196-211 (2010).

17. Jing, Z. et al. Sound velocity of Fe–S liquids at high pressure: Implications for the Moon's molten outer core. *Earth Planet. Sci. Lett.* **396**, 78-87 (2014).

18. Jenkins, D. M., Corona, J. C., Bassett, W. A., Mibe, K. & Wang, Z. Compressibility of synthetic glaucophane. *Phys. Chem. Miner.* **37**, 219-226 (2010).

19. Ross, J. A., Matteo, A. & Javier, G.-P. EosFit7c and a Fortran module (library) for equation of state calculations. *Zeitschrift für Kristallographie - Crystalline Materials* **229**, 405-419 (2014).

20. Murnaghan, F. FD Murnaghan, Am. J. Math. 59, 235 (1937). *Am. J. Math.* **59**, 235 (1937).

21. Ishida, K. Infrared spectra of alkali amphiboles of the glaucophane-riebeckite series and their relation to chemical composition. *Mineral. J.* **15**, 147-161 (1990).

22. Gilletillet, P., Reynard, B. & Tequi, C. Thermodynamic properties of glaucophane new data from calorimetric and spectroscopic measurements. *Phys. Chem. Miner.* **16**, 659-667 (1989).

23. Madon, M. & Price, G. D. Infrared spectroscopy of the polymorphic series (enstatite, ilmenite, and perovskite) of $MgSiO_3$, $MgGeO_3$, and $MnGeO_3$. *J. Geophys. Res. Solid Earth* **94**, 15687-15701 (1989).

24. Engdahl, E. R., van der Hilst, R. & Buland, R. Global teleseismic earthquake relocation with improved travel times and procedures for depth determination. *Bulletin of the Seismological Society of America* **88**, 722-743 (1998).

25. Weston, J., Engdahl, E. R., Harris, J., Di Giacomo, D. & Storchak, D. A. ISC-EHB:

reconstruction of a robust earthquake data set. *Geophysical Journal International* **214**, 474-484 (2018).

26. Engdahl, E. R. et al. ISC-EHB 1964–2016, an Improved Data Set for Studies of Earth Structure and Global Seismicity. *Earth and Space Science* **7**, e2019EA000897 (2020).

27. White, R. S., McKenzie, D. & O'Nions, R. K. Oceanic crustal thickness from seismic measurements and rare earth element inversions. *J. Geophys. Res. Solid Earth* **97**, 19683-19715 (1992).

28. Geissler, W. H., Jokat, W., Jegen, M. & Baba, K. Thickness of the oceanic crust, the lithosphere, and the mantle transition zone in the vicinity of the Tristan da Cunha hot spot estimated from ocean-bottom and ocean-island seismometer receiver functions. *Tectonophysics* **716**, 33-51 (2017).

29. Fowler, C. M. R. *The Solid Earth: An Introduction to Global Geophysics*, 2nd edn. 285-288 (Cambridge Univ. Press, Cambridge (2005).

30. Wyllie, P. J. *The Dynamic earth: textbook in geosciences*. 139-165 (John Wiley & Sons, Inc, New York (1971).

31. Carlson, R. L. & Herrick, C. N. Densities and porosities in the oceanic crust and their variations with depth and age. *J. Geophys. Res. Solid Earth* **95**, 9153-9170 (1990).

32. Ronov, A. B. & Yaroshevsky, A. A. Chemical Composition of the Earth's Crust. (ed Hart, P. J.) in *The Earth's Crust and Upper Mantle*, vol. 13. 37-57 (Am. Geophys. Union, Washington, 1969).

33. Charette, M. A. & Smith, W. H. The volume of Earth's ocean. *Oceanography* **23**, 112-114 (2010).

34. Mackenzie, F. T. & Garrels, R. M. Chemical mass balance between rivers and oceans. *Am. J. Sci.* **264**, 507-525 (1966).

35. Schmidt, M. W. & Poli, S. Experimentally based water budgets for dehydrating slabs and consequences for arc magma generation. *Earth Planet. Sci. Lett.* **163**, 361-379 (1998).

36. Faccenda, M. Water in the slab: A trilogy. *Tectonophysics* **614**, 1-30 (2014).

37. van Keken, P. E., Hacker, B. R., Syracuse, E. M. & Abers, G. A. Subduction factory: 4. Depth-dependent flux of H_2O from subducting slabs worldwide. *J. Geophys. Res. Solid Earth* **116**, B01401 (2011).

38. Molnar, P., Freedman, D. & Shih, J. S. F. Lengths of intermediate and deep seismic zones and temperatures in downgoing slabs of lithosphere. *Geophysical Journal International* **56**, 41-54 (1979).

39. McKenzie, D. P. Speculations on the Consequences and Causes of Plate Motions. *Geophysical Journal International* **18**, 1-32 (1969).

40. Kirby, S. H., Durham, W. B. & Stern, L. A. Mantle Phase Changes and Deep-Earthquake Faulting in Subducting Lithosphere. *Science* **252**, 216 (1991).

41. England, P., Engdahl, R. & Thatcher, W. Systematic variation in the depths of slabs beneath arc volcanoes. *Geophysical Journal International* **156**, 377-408 (2004).
